# *Limosilactobacillus reuteri* L26 Biocenol^TM^ and its exopolysaccharide: Their influence on rotavirus-induced immune molecules in enterocyte-like cells

**DOI:** 10.17221/106/2023-VETMED

**Published:** 2024-05-27

**Authors:** Petra Schusterova, Dagmar Mudronova, Katarina Loziakova Penazziova, Vanda Hajduckova, Tomas Csank

**Affiliations:** Department of Microbiology and Immunology, University of Veterinary Medicine and Pharmacy in Košice, Košice, Slovak Republic

**Keywords:** innate antivirus immunity, lactic acid bacteria, RVA OSU 6, type III interferon

## Abstract

This study aimed to evaluate the immunomodulatory effect of the probiotic *Limosilactobacillus reuteri* L26 Biocenol^TM^ (L26) and its purified exopolysaccharide (EPS) with respect to antiviral innate immune response. In our experiment, we used porcine epithelial IPEC-J2 cells as a model of the intestinal barrier in a homologous infection by porcine *Rotavirus* *A* strain OSU6 (RVA). The production of selected molecules of non-specific humoral immunity was evaluated at the mRNA level. The EPS alone significantly increased the level of interferon λ3 (IFN-λ3) mRNA in the non-infected IPEC-J2 cells (*P* < 0.001). We also tested whether the treatment of IPEC-J2 cells by L26 or EPS influences the replication of RVA by virus titration and real-time PCR. We found that a pre-treatment in combination with subsequent continuous stimulation has no influence on the RVA replication. However, both treatments significantly decreased the RVA-induced production of IFN-λ3 (*P* < 0.05) and the “SOS” cytokine interleukin 6 (IL-6; *P* < 0.01), already at the transcription level. In addition, the EPS treatment resulted in significantly increased IL-10 mRNA in the RVA-infected cells. In summary, we assume an immunoregulatory potential of *L. reuteri* L26 Biocenol^TM^ and its EPS in the local intestinal antiviral immune response.

Probiotics and their products have attracted the attention of scientists for their beneficial effects on human and animal health. Lactobacilli are one of the major groups of Gram-positive immunomodulatory lactic acid bacteria (LAB) – immunobiotics and are generally recognised as safe (GRAS).

The gastrointestinal tract is one of the pathogenic microorganisms’ most important entry sites. A balance between physiological and immunological processes maintains its barrier function. Intestinal viral infections often alter that balance, disrupting the epithelial barrier integrity and causing diarrhoea. One of the most common causes of viral diarrhoea is *Rotavirus A* (RVA). Rotavirus infections cause intestinal malabsorption and maldigestion by destroying the terminally differentiated enterocytes at the tips of the intestinal villi (MacLachlan and Dubovi 2011). This results in watery diarrhoea, dehydration, anorexia, and weight loss with high mortality and morbidity in piglets.

Several research groups reported the antiviral effect of immunomodulatory LAB or their products in intestinal epithelial cells (Liu et al. 2010; Albarracin et al. 2017; Kanmani et al. 2018; Mizuno et al. 2020). The *Limosilactobacillus reuteri* strain L26 Biocenol^TM^ (CCM 8616) was originally isolated from the gut content of a healthy suckling piglet and its exopolysaccharide (EPS) was characterised as a high molecular weight d-glucan polysaccharide with (1→3) and (1→6) glycosidic linkages (Ksonzekova et al. 2016). Due to the significant immunoregulatory potential of probiotics or their products in intestinal mucosal immunity (Ksonzekova et al. 2016; Karaffova et al. 2017; Mudronova et al. 2018; Kanmani et al. 2018; Kim et al. 2018; Mizuno et al. 2020), they are increasingly used as oral vaccine adjuvants or carriers.

Here, we report the results of the study of the immunomodulatory and antiviral capacity of *L. reuteri* L26 and its purified EPS under *in vitro* conditions using porcine IPEC-J2 enterocyte cells and the RVA OSU6 strain.

## MATERIAL AND METHODS

### IPEC-J2 cell line cultivation

The IPEC-J2 cell line was kindly provided by Juan José Garrido (Department of Genetics and Animal Breeding, University of Cordoba, Spain). The cells were several times passaged in our laboratory and tested negative for mycoplasma (Young et al. 2010). The composition of the complete IPEC-J2 cultivation medium was as follows: DMEM/F-12 (PAN-Biotech, Aidenbach, Germany), 5% foetal bovine serum (FBS, PAN-Biotech), 2% l-glutamine (PAN-Biotech), 50 μg/ml of gentamicin (Biowest, Nuaillé, France), 5 ng/ml of epidermal growth factor (BD Biosciences, San José CA, USA), 10 μg/ml of insulin, 10 μg/ml of transferrin and 10 ng/ml of selenium (ITS, Lonza, Valais, Switzerland). The cells were cultivated at 37 °C in a humidified 5% CO_2_ atmosphere.

### *L. reuteri* L26 strain and its EPS

*L. reuteri* L26 Biocenol^TM^ (CCM 8616) was stored in a Microbank^TM^ cryoprotective medium (Pro-Lab Diagnostics, Austin, Texas) at –70 °C. The bacteria were inoculated on a de Man, Rogosa and Sharpe (MRS) agar (Merck, Darmstadt, Germany) and anaerobically cultivated at 37 °C. After 48 h, the bacterial suspension was prepared in the MRS broth and cultivated overnight at 37 °C with continuous stirring. The bacteria in the overnight broth culture were quantified by serial 10-fold dilution and expressed as the number of colony forming units per millilitre (CFU/ml). Details of the isolation, purification, and characterisation of chemical properties of the used EPS is described in Ksonzekova et al. (2016). Briefly, the EPS were isolated using ethanol extraction, and purified with proteinase K and trichloroacetic acid to remove the proteins or peptides. Finally, the supernatant with EPS was dialysed to remove salts and other components in order to obtain pure EPS. Lyophilised EPS was dissolved in sterile deionised water.

### Virus cultivation

The porcine RVA OSU6 (CAPM V-334) was obtained from the Collection of Animal Pathogenic Microorganisms (Veterinary Research Institute, Brno, Czech Republic). Prior to infection with MA104 (ECACC 85102918), the virus was treated with trypsin (10 μg/ml, 37 °C, 40 min). The cell monolayer was three times washed with Dulbecco’s Modified Eagle Medium (DMEM)/F12 without FBS and infected with the trypsin-treated virus. After one-hour of adsorption at 37 °C, the inoculum was removed, and the cells were washed with DMEM/F12 without FBS. The cells were further cultivated in a cultivation medium without FBS. When the complete cytopathic effect (CPE) was developed, the medium was collected, clarified by centrifugation at 4 000 × *g* for 10 min at 4 °C and the aliquots of the virus stock were stored at –80 °C.

### Experimental design

First, we analysed the immunomodulatory activity of *L. reuteri* L26 and its EPS. The cells were seeded at a density of 1 × 10^5^/cm^2^ per well in a collagen-treated (10 μg/cm^2^; Collagen type I from rat tail; Sigma-Aldrich, St. Louis, USA) 24-well plate (Greiner bio-one) and cultivated overnight at 37 °C in a humidified 5% CO_2_ atmosphere. After thorough washing with DMEM/F-12, the cells were stimulated with 1.6 × 10^5 ^CFU/ml of *L. reuteri* L26, or with 1 mg/ml of EPS. To continuously stimulate the cells with bacteria, we used a filtered (0.22 μm filters, Merck) FBS-free cultivation medium without gentamicin. The cells were stimulated for 21 hours.

Second, we focused on the effect of the bacterial and EPS stimulation on the virus replication and the mRNA level of the selected cytokines. Here, after five hours of stimulation, the medium was discarded, and the cells were infected with 100 μl (3.9 × 10^6^ PFU/ml) of the trypsin-treated RVA in an FBS-free cultivation medium resulting in MOI 2. Ensuring continuous stimulation, the virus inoculum contained 1.6 × 10^5 ^CFU/ml *L. reuteri* L26 or 1 mg/ml of EPS. After one hour of adsorption at 37 °C, 750 μl of filtered FBS-free cultivation medium without gentamicin was added and the cells were further incubated for 15 hours. Cell viability was assessed by light microscopy with minimal cell damage detected [Electronic Supplementary Material (ESM) Figure S1 and S2]. Virus infection was assessed by fluorescence microscopy (ESM Figure S3).

In both experiments after incubation, the medium from each well was collected, centrifuged (16 000 × *g*, 5 min, 4 °C) and stored at –80 °C for virus titration and the cells were used for RNA extraction.

### Virus titration

The RVA OSU6 titration in the stock and in the cultivation media of infection groups was carried out on MA104 cells. The cells were seeded on 96-well plates (Greiner bio-one, Kremsmünster, Austria) at a density of 2.5 × 10^4^/well and cultivated overnight in a cultivation medium. The virus stock was serially 10-fold diluted in a cultivation medium without FBS and supplemented by trypsin to a final concentration of 0.1 μg/ml. Each dilution was tested in quadruplicate. The virus titre was calculated using Spearman’s method and expressed as 50% tissue culture infective dose per millilitre (TCID_50_/ml). For the calculation of the multiplicity of infection (MOI), TCID_50_/ml was converted to plaque-forming units per millilitre (PFU/ml) by multiplying by 0.7 (Science Gateway n.d.).

### RNA extraction and cDNA synthesis

One microgram of the total RNA extracted by NucleoZOL (Macherey-Nagel, Düren, Germany) was treated with DNase I (Thermo Scientific, Waltham, MA, USA) and used for the cDNA synthesis with a LunaScript^TM^ RT SuperMix kit (NEB, Ipswich, Massachusetts, USA).

### Intracellular virus RNA quantification

The virus RNA quantification in the cells was performed using the Luna^®^ Universal qPCR MasterMix (NEB) according to the manufacturer’s instructions containing 0.25 μM of primers [see Electronic Supplementary Material (ESM) – Table S1]. The thermal profile was as follows: initial denaturation at 95 °C for 1 min; 45 cycles at 95 °C for 15 s and at 52.5 °C for 30 s followed by a melting curve analysis. The calibration curve (*R*^2^ = 0.995) was prepared from the 10-fold diluted virus stock with a known PFU/ml.

### Gene expression analysis

The analysis of interferon λ3 (IFN-λ3), IL-6, IL-18, IL-10, transforming growth factor β (TGF-β) and the β-actin gene expression was performed by a Luna^®^ Universal qPCR MasterMix (NEB) containing 40 ng of cDNA using the primers listed in ESM Table S1. The reaction runs at the conditions: at 95 °C for 1 min; 45 cycles at 95 °C for 15 s and at 55 °C or 60 °C for 30 s, followed by melting curve analysis. Each assay included no template control. The reaction efficiency of each gene reached 95 ± 5%. The relative normalised expression was calculated by the 2^–ΔΔCt^ method using CFX96 Manager Software (BioRad) compared to the β-actin reference gene.

### Statistical analysis

The results were expressed as the mean ± standard deviation (SD, *n* = 3). The data were evaluated using the GraphPad Prism v3.00 software by a one-way analysis of variance (ANOVA) followed by Tukey’s multiple comparison test.

## RESULTS

### The immunomodulatory activity of *L. reuteri* L26 and its EPS on IPEC-J2 cells

**Figure 1:**
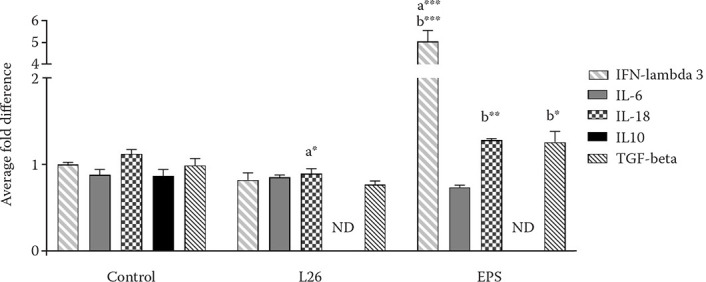
Gene expression profile of the IPEC-J2 cells after 21 h of stimulation with live *Limosilactobacillus reuteri* L26 (L26) or its exopolysaccharide (EPS) The results are expressed as the mean ± SD. Significantly different: a – compared to the control; b – compared to the L26 group (*P < 0.05; **P < 0.01; ***P < 0.001). Control – IPEC-J2 cells without stimulation ND = not detected

Figure 1 depicts the immunomodulatory activity of *L. reuteri* L26 or its EPS on IPEC-J2 cells. There was no significant change observed in the IL-6 mRNA level. Messenger RNA of IL-10 was detected only in the control cells. When compared to the control group, the L26 treatment led to a significantly lower IL-18 mRNA level, but the EPS treatment resulted in a moderate increase. The most considerable change in the gene expression was observed after the EPS treatment, where the IFN-λ3 mRNA level reached approximately a 5-fold (5.1 ± 0.87) increase in comparison with L26 and the control group.

### Effect of *L. reuteri* L26 and its EPS on the RVA replication and on the RVA-induced innate immune molecules

**Figure 2:**
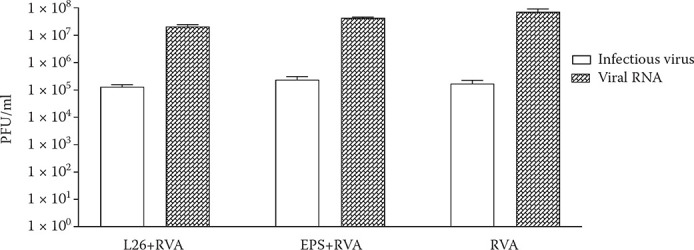
Quantification of the extracellular RVA OSU6 and intracellular viral RNA The cells were stimulated with *Limosilactobacillus reuteri* L26 (L26+RVA) or its exopolysaccharide (EPS+RVA) for 5 h before infection. RVA-infected cells (one-hour adsorption) were additionally cultivated for 15 h with continuous stimulation. The absolute quantification of the viral RNA in the IPEC-J2 cells was carried out based on a standard curve prepared by a serial 10-fold dilution of the virus stock. Empty bars – quantification of the extracellular infectious virus by titration, grey bars – quantification of the intracellular viral RNA by real-time polymerase chain reaction (PCR). RVA – the cells were cultivated under the same conditions and were not stimulated only infected

**Figure 3:**
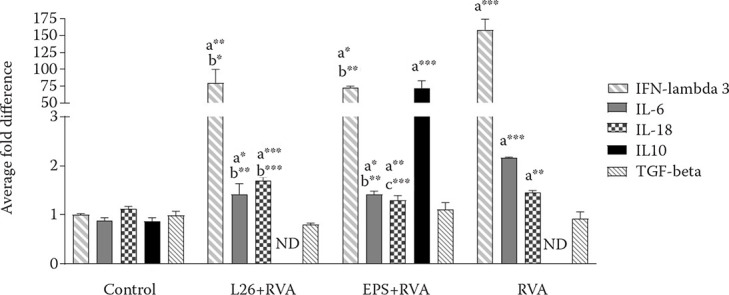
The effect of *Limosilactobacillus reuteri* L26 (L26) and its exopolysaccharide (EPS) on the gene expression of the cytokines in the RVA OSU6 (MOI = 2) infected IPEC-J2 cells The cells were continuously stimulated with L26 or EPS during the whole experiment for 21 hours. The results are expressed as the mean ± SD. Significantly different: a – compared to the control, b – compared to the RVA, c – compared to the L26 + RVA group (*P < 0.05; **P < 0.01, ***P < 0.001). Control – IPEC-J2 cells without stimulation or infection. EPS+RVA – cells were stimulated with EPS and RVA infected; L26+RVA – cells were stimulated with L26 and RVA infected; RVA – the cells were not stimulated, only RVA infected ND = not detected

The continuous stimulation of the IPEC-J2 cells with *L. reuteri* or its EPS does not influence the virus replication (Figure 2). The TGF-β mRNA level showed no significant changes after the L26 or EPS treatments in the infected cells (Figure 3). As expected, the mRNA level of IFN-λ3 was significantly higher in all the infected groups (up to 158.7-fold) in comparison with the control group. However, when compared to the RVA group, the treatment either with *L. reuteri* L26 (80.1 ± 34.36; *P* < 0.05) or its EPS (72.5 ± 4.49; *P* < 0.01) resulted in a significantly lower IFN-λ3 mRNA level. In comparison to the control group, the RVA infection caused a 2.2-fold increase in the IL-6 mRNA level. However, the L26 and EPS treatment caused the mRNA decrease. IL-18 reached the significantly highest mRNA level in comparison with each experimental group (*P* < 0.001) after treatment with *L. reuteri*. Similarly, to the first experiment, IL-10 mRNA was not detected in the RVA and L26+RVA groups. On the other hand, the EPS stimulation and RVA infection resulted in a 72.3-fold increase in the IL-10 mRNA level.

## DISCUSSION AND CONCLUSION

The characterisation of the gene expression of selected epithelial and innate immune-related markers showed that the IPEC-J2 cell line is a valuable model for host-probiotic/pathogen interaction studies in the porcine model (Mariani et al. 2009). The present study analysed the immunomodulatory properties of *L. reuteri* L26 Biocenol^TM^ and its purified EPS on IPEC-J2 cells. Mudronova et al. (2018) supposed that the lactobacilli-produced EPSs are one of the most important immunomodulatory products of the L26 strain, while the produced organic acids by this strain contribute to its antimicrobial potential. According to this assumption, we observed a 5-fold increase in the IFN-λ3 mRNA level after the EPS treatment of the IPEC-J2 cells, but not in the L26 group. It is important to note that the used lactobacilli were cultivated in a standard MRS and IPEC-J2 cultivation medium and probably produced a small amount of EPS (Ksonzekova et al. 2016). The immunostimulatory effect of EPS needs to be confirmed by further studies also at the protein level. IFN-λ3 is a type III IFN that primarily acts on the epithelial cells and elicits a first-line antiviral response (Sommereyns et al. 2008; Sang et al. 2010; Deng et al. 2022). The gene expression of IFN-λ3 was also observed in IPEC-J2 cells treated with poly(I:C), which mimics viral dsRNA (Sang et al. 2010) and induces a similar antiviral immunotranscriptomic response as RVA does (Albarracin et al. 2017). Li et al. (2019) compared the transcriptional profile of type I and type III IFNs in the IPEC-J2 cells and showed that pre-treatment with IFN-λ3 induces a non-redundant antiviral response through the strong stimulation of most IFN stimulated genes (ISGs). Deng et al. (2022) found that IFN-λ3 was superior to IFN-α in inhibiting rotavirus replication in epithelial cells, which correlated with its better stimulatory effect on the ISG expression. Interestingly, co-treatment with IFN-λ3 and IFN-α did not enhance this antiviral activity (Deng et al. 2022). Type III IFNs are important antiviral molecules at the epithelial barriers, possibly due to tissue- and cell-specific receptor distribution. Lactic acid bacteria may have different influences on virus replication. The administration of *L. reuteri* L26 to gnotobiotic mice resulted in the stimulation of the local intestinal immunity and decreased the amount of porcine circovirus type 2 (PCV-2) in the faeces and ileum (Karaffova et al. 2017). On the other hand, *Lactobacillus acidophilus* and *L. reuteri* colonisation did not reduce shedding and diarrhoea after a human rotavirus strain Wa infection in neonatal pigs (Zhang et al. 2008). Liu et al. (2010) reported that the pre-treatment of IPEC-J2 cells with *L. acidophilus* increased the virus replication of RVA OSU6. In the study of Leblanc et al. (2022), a similar porcine enterocyte (IPEC-J2 cells)-probiotics-virus *in vitro* platform was used in combination with flow cytometry. They found that the longer pre-treatment (16 h) of the enterocytes or virus with probiotics (*Bifidobacterium longum* R0175, *B. animalis* subsp. *lactis* A026, *Lactobacillus plantarum* 299V) inhibit the rotavirus infection. In the present study, we did not observe any influence of either the EPS or L26 on the replication of RVA OSU. Taken together with previous studies, different *Lactobacillus* species may have different effects on the virus replication and further work is necessary in this field.

The influence of *L. reuteri* L26 and its EPS on the RVA-induced innate immune response in IPEC-J2 cells was also analysed. The rotavirus infection induces gene expression or production of several immunologically important molecules in intestinal cells, for example IL-6 (Liu et al. 2010), IL-8 (Sheth et al. 1996; Liu et al. 2010), IL-2, IL-10, TNF-α, INF-γ, MIP-1α, MCP-1 (Huang et al. 2020), IFN-λ, IL-18 and IL-22 (Xue et al. 2017). In this experiment, the RVA infection itself increased the IFN-λ3, IL-6 and IL-18 mRNA, but the bacterial and EPS treatment lowered the RVA-stimulated IFN-λ3 and IL-6 mRNA levels. This finding is in concordance with a previous study, where *L. reuteri* and its EPS significantly decreased the expression of the pro-inflammatory IL-1β and the IL-6 cytokines in porcine enterocyte-like cells after infection with the enterotoxigenic *Escherichia coli* (Ksonzekova et al. 2016). However, Kanmani et al. (2018) reported a decrease in the IL-6 mRNA in EPS treated poly(I:C)-activated enterocyte-like cells.

Regarding IL-18, we found that the L26 stimulation with the subsequent RVA OSU6 infection slightly increased the IL-18 mRNA level compared to each experimental group (*P* < 0.001). The gene expression of IFN-λ3 or IL-18 is regulated by IL-22 in a STAT3-dependent manner, which supports the synergistic feedback loop between these cytokines (Xue et al. 2017). In our experiment, IL-22 mRNA in the IPEC-J2 cells was not detected (data not shown). Zhang et al. (2014) reported preventive and therapeutic effects of IL-18 in combination with IL-22. They found that TLR5 or NLRC4 receptor activation in mouse dendritic cells *in vivo* results in increased IL-22 or IL-18 production and the immediate elimination of RVA-infected intestinal epithelial cells.

IL-10 plays a role in immunoregulation or immunosuppression. In the present study, the IL-10 mRNA expression was found only in the control groups and in the EPS treated infected cells. Differences between the EPS treatment and the live lactobacilli treatment could probably be due to the above-mentioned cultivation conditions, which may affect the production of the EPS of the used bacteria. This result shows that the EPS of *L. reuteri* itself may have an immunoregulatory or immunosuppressive effect. Information about the expression of this anti-inflammatory cytokine under similar experimental conditions are limited, but *in vivo* studies using other lactic acid bacteria support this finding. *Lactobacillus delbrueckii* subsp. *lactis* CRL581 significantly increased the intestinal IL-10 detected by an enzyme-linked immunosorbent assay (ELISA) either itself or in poly(I:C) challenged mice (Elean et al. 2020). Also, increased intestinal and serum levels of IL-10 were observed in *L. rhamnosus* and *Lactiplantibacillus plantarum* treated mice challenged with poly(I:C) (Tada et al. 2016).

In conclusion, neither treatment with the *L. reuteri* strain L26 nor its EPS influenced the RVA replication in the IPEC-J2 cells. Both treatments followed by a virus infection resulted in a decrease in the RVA-induced IFN-λ3 and IL-6 mRNA levels. Based on the present (with the RVA infection) and our earlier [with an Enterotoxigenic *E. Coli* (ETEC) infection] *in vitro* study, we assume that *L. reuteri* L26 Biocenol^TM^ and especially its EPS may have an important role in the regulation of the local gut innate immune response against intestinal pathogens. However, further studies are necessary to obtain a deeper insight into the antiviral potential of the probiotics and their products.
