# Supplementary material for: Limosilactobacillus reuteri L26 BiocenolTM and its exopolysaccharide: Their influence on rotavirus-induced immune molecules in enterocyte-like cells
Source: Vet Med (Praha). 2024 May 27;69(5):169–76. doi: 10.17221/106/2023-VETMED (PMC11148706; doi:10.17221/106/2023-VETMED)
Supplement: Supplementary Files [file VETMED-69-05-123106-s001.pdf]

https://doi.org/10.17221/106/2023-VETMED

Limosilactobacillus reuteri L26 Biocenol™ and its exopolysaccharide: Their influence on rotavirus-induced immune molecules in enterocyte-like cells

*Petra Schusterova, Dagmar Mudronova, Katarina Loziakova Penazziova, Vanda Hajduckova, Tomas Csank**

Department of Microbiology and Immunology, University of Veterinary Medicine and Pharmacy in Košice, Košice, Slovak Republic

Corresponding author: tomas.csank@uvlf.sk

The authors are fully responsible for both the content and the formal aspects of the electronic supplementary material. No editorial adjustments were made.

Electronic Supplementary Material (ESM)

Table S1. Characteristics of the used primers

Figure S1. IPEC-J2 cells in the negative control before harvesting the cultivation medium and the cells

Figure S2. IPEC-J2 cells showing the beginning of the cytopathic effect in the RVA group at the time of harvesting of the cultivation medium and the cells

Figure S3. Immunofluorescent detection of RVA OSU6 in the IPEC-J2 cells in the RVA group before harvesting the cultivation medium and the cells (× 400 total magnification)

https://doi.org/10.17221/106/2023-VETMED

Table S1. Characteristics of the used primers

Genes	Sequence (5'→3')	T_a (°C)	Conc. (μM)	Source
β-actin	F: CATCACCATCGGCAACGA<br>R: GCGTAGAGGTCCTTCCTGATGT	55<br>60	0.25	Moue et al. (2008)
IFN-λ3	F: TGGCCCAGTTCAAGTCTCTG<br>R: TGCAGTTCCAGTCCTCCAAG	55	0.5	this study
IL-6	F: TGGATAAGCTGCAGTCACAG<br>R: ATTATCCGAATGGCCCTCAG	60	0.25	Moue et al. (2008)
IL-18	F: AGCCGTGTTTGAGGATATGCC<br>R: GGTTACTGCCAGACCTCTAGTG	55	0.25	this study
IL-10	F: ACTTCCCAACCAGCCTGC<br>R: TCAGCAACAAGTCGCCCAT	55	0.25	this study
TGF-β	F: CACGTGGAGCTATACCAGAA<br>R: TCCGGTGACATCAAAGGACA	60	0.25	Moue et al. (2008)
RVA-VP7	F: CAACTGCACCACAAACTGAAAGA<br>R: CTCGGTAATAAAAGGCAGCAGAA	52.5	0.25	Xue et al. (2017)

REFERENCES

Moue M, Tohno M, Shimazu T, Kido T, Aso H, Saito T, Kitazawa H. Toll like receptor 4 and cytokine expression involved in functional immune response in an originally established porcine intestinal epitheliocyte cell line. Biochim Biophys Acta. 2008 Feb;1780(2):134-44.

Xue M, Zhao J, Ying L, Fu F, Li L, Ma Y, Shi H, Zhang J, Feng L, Liu P. IL-22 suppresses the infection of porcine enteric coronaviruses and rotavirus by activating STAT3 signal pathway. Antiviral Res. 2017 Jun;142:68-75.

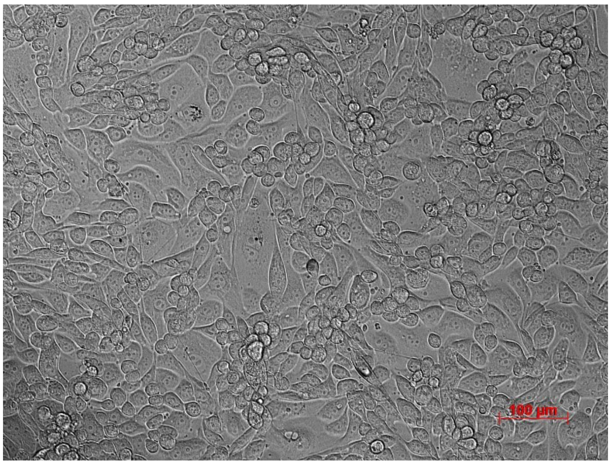

Figure S1. IPEC-J2 cells in the negative control before harvesting the cultivation medium and the cells

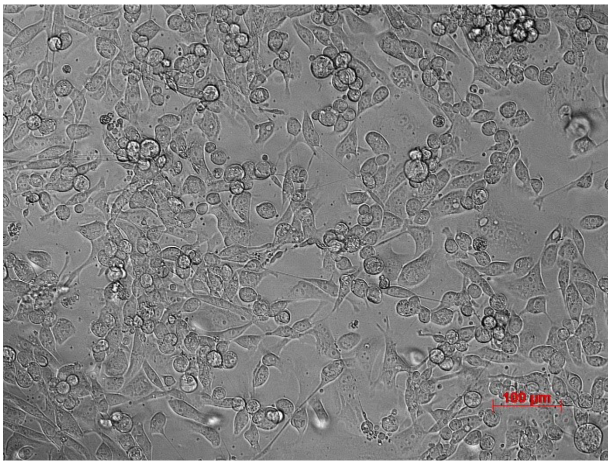

Figure S2. IPEC-J2 cells showing the beginning of the cytopathic effect in the RVA group at the time of harvesting of the cultivation medium and the cells

https://doi.org/10.17221/106/2023-VETMED

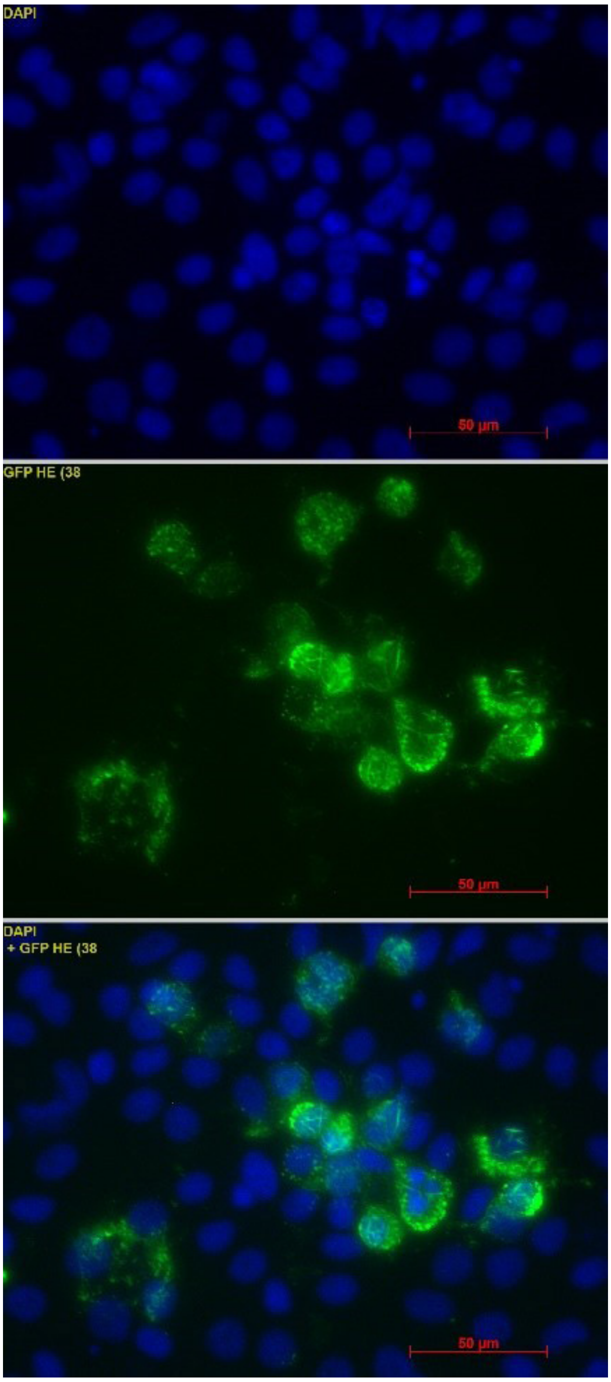

Figure S3. Immunofluorescent detection of RVA OSU6 in the IPEC-J2 cells in the RVA group before harvesting the cultivation medium and the cells (× 400 total magnification)

RVA OSU6 infected IPEC-J2 cells in the RVA group on the coverslips were fixed with ice-cold methanol for 10 min at –20 °C. After fixation, the cells were washed twice with PBS containing 0.05% Tween 20 (TPBS). After aspiration of the washing medium, the primary mouse monoclonal antibody to RVA p42 (MyBioSource, USA) at a dilution of 1 : 500 in TPBS was added for 1 h at 37 °C. The coverslips were then washed three times with TPBS and a secondary goat anti-mouse immunoglobulin antibody conjugated to Alexa Fluor 488 was added (1§: 750; Abcam, UK) for 30 min at 37 °C. Cell nuclei were stained with DAPI (1 : 1 000; Sigma Aldrich, Germany) for 5 min at 37 °C. Immunofluorescence was monitored using an AxioObserver Z.1 inverted fluorescence microscope (Carl Zeiss, Germany)
